# Neural Resolution of Formant Frequencies in the Primary Auditory Cortex of Rats

**DOI:** 10.1371/journal.pone.0134078

**Published:** 2015-08-07

**Authors:** Christian Honey, Jan Schnupp

**Affiliations:** 1 Berlin, Germany; 2 Department of Physiology, Anatomy and Genetics, University of Oxford, Oxford, United Kingdom; University of Chicago, UNITED STATES

## Abstract

Pulse-resonance sounds play an important role in animal communication and auditory object recognition, yet very little is known about the cortical representation of this class of sounds. In this study we shine light on one simple aspect: how well does the firing rate of cortical neurons resolve resonant (“formant”) frequencies of vowel-like pulse-resonance sounds. We recorded neural responses in the primary auditory cortex (A1) of anesthetized rats to two-formant pulse-resonance sounds, and estimated their formant resolving power using a statistical kernel smoothing method which takes into account the natural variability of cortical responses. While formant-tuning functions were diverse in structure across different penetrations, most were sensitive to changes in formant frequency, with a frequency resolution comparable to that reported for rat cochlear filters.

## Introduction

Pulse-resonance sounds (PRSs) are the backbone of most animals vocalization [1] and vowels in human speech are a prominent member of this sound class. They are richer and more ecologically relevant than the pure tones or noise bursts more commonly used in physiological studies, but they remain simple enough to be described by a relatively small number of parameters. In PRSs, a periodic source produces a regular, click-train like input to a set of resonators. The sound consequently consists of a series of harmonics of unequal amplitude, and the harmonics closest to the peak resonant frequencies (the so-called “formant frequencies”) are largest. Most mammalian vocal sounds, including vowels in human speech, are examples of PRSs, in which the fundamental frequency of the harmonics is determined by the glottal pulse rate emanating from the larynx, and the formant frequencies depend on the size of resonant cavities in the vocal tract. Thus, the pitch of such vocal sounds depends on their fundamental frequency, but the “identity” of the sound is mostly recognized from its formant frequencies [2].

Given the ecological importance of this class of sound, responses of cortical neurons to natural or artificial vocal sounds have been studied in gerbils [3], ferrets [4–6] monkeys [7], guinea pigs [8,9] and humans [10].

However, most studies so far have focused on the strength of the neural response as a function of large changes in stimulus parameters such as fundamental or formant frequencies. No study to date has used PRSs to explore the sensitivity of cortical responses to small changes in the position of formant peaks, or charted the distribution of such sensitivity. Studying these aspects of tuning to acoustic features of complex sounds may be revealing when one seeks to understand the role particular parts of the auditory pathway play in phonetic tasks. Consider, for example, that neurons involved in recognizing speech sound categories are unlikely to discriminate acoustic features at the very physiological limits of frequency selectivity, so that phonetically unimportant "within phone category" variability of the acoustic parameters does not lead to radically different neural activation patterns [11]. Consequently one might expect to see neural tuning curves to broaden somewhat to reflect acoustic category distributions as one ascends along a neural pathway involved in phonetic processing. To what extent this expectation is borne out is not entirely clear. Cortical pure tone tuning curves are normally considerably broader than those seen at the lowest stations of the lemniscal auditory pathway. However, tuning curves or spectro-temporal receptive fields of cortical neurons often predict the same neurons' responses to complex sounds poorly [12–14]. Consequently it may not be advisable to try to understand the neural representation of the formants of pulse-resonance sounds by extrapolating from pure tone tuning functions.

In this study we used simple, synthetic PRSs with two formant peaks (2FPRSs) to map out cortical ‘formant receptive fields’ and to estimate the frequency resolution at which these receptive fields operate. We chose two formant sounds because animal vocal sounds are often characterized by particular formant combinations. Indeed, the identity of human vowels appears to be determined by their position in “F1/F2 formant space” [15], where F1, F2 refer to the lowest and second lowest formant frequencies. We also wanted to keep the stimuli simple and generic, choosing sounds that are just complex enough to span a “formant space” and to mimic the characteristic pulse-resonance nature of many natural sounds, but simple enough to avoid adding unnecessary complexity to the stimuli.

We used rats as a model species as they are known to discriminate and categorize PRSs, such as human speech sounds, at high levels of performance both in their behavior [16,17] as well as in their cortical physiological responses [18].

Cortical responses to sounds exhibit considerable trial-to-trial variability [19,20], and both for the experimental observer and for the brain itself the question whether a sensory neuron is able to discriminate between two similar stimuli essentially reduces to the question whether observed response differences are due to more than just the level of chance variability which might be expected if the same stimulus was presented twice. In order to achieve statistically robust estimates of the “resolving power” of neural formant tuning functions in the presence of such “physiological noise” we devised and applied a novel measure, based on kernel smoothing [21].

Our results reveal highly variable formant receptive fields structures across the cortical population, but despite the diversity and variability of the observed response patterns, most neural responses nonetheless appear to resolve formant frequencies with a similar, high resolution of 0.1 octaves or better. Formant resolution tended to be very similar along anatomically neighboring recording sites, but varied considerably across different penetrations.

## Materials and Methods

### Surgery

All animal procedures were approved by Oxford University’s “Committee on Animal Care and Ethical Review” and performed under license from the UK Home Office in accordance with the Animal (Scientific Procedures) Act 1986. Four naive adult female rats (Sprague-Dawley, 240–265 g), were used for extracellular recordings from A1 under terminal general anesthesia. Anesthesia was induced by intraperitoneal injection of ketamine (Ketaset; Fort Dodge Animal Health, Overland Park, Kansas, USA) and medetomidine (Domitor, Pfizer, Walton Oaks, Surrey, UK), and maintained with intraperitoneal infusions of ketamine, medetomidine and butorphanol tartrate (Torbugesic; Fort Dodge Animal Health, Overland Park, Kansas, USA), at typical rates of 4.0 mg/kg/h, 16 μg/kg/h and 0.5 mg/ kg/h, respectively. Medical oxygen was administered at 0.5l/h to the animals’ snout. The animals' core temperature was monitored and kept at 38°C using a rectal temperature probe and homeothermic blanket. Heart rate was monitored throughout the experiment and the depth of anesthesia was confirmed with regular paw and eye-lid reflex tests.

After inducing anesthesia, the skull was exposed and a 3–4 mm diameter craniotomy was performed over auditory cortex at coordinates 5 mm anterior of bregma, 7.2 mm lateral to the right of the midline, and 4.5 mm ventral from the vertex [22]. The coordinates were chosen to center above primary auditory cortex, and were adjusted slightly according to the animals’ individual size. An additional small craniotomy was made over left frontal cortex and a silver wire was placed into the epidural space as an electrical reference for recordings.

### Electrophysiology

The animals were placed in a soundproofed recording chamber and extracellular signals were recorded using Neuronexus (AnnArbour, Michigan) A-style single-shank silicon probes (16 recording sites at 100 μm spacing, or 32 channels at 50 μm spacing, 177 μm^2^ surface area) and TDT (Tucker Davies Technologies, Alachua, FL) RA16 measuring amplifiers and RZ5 digital signal processors. Neural signals were recorded with full bandwidth at 24 kHz sampling rate and monitored online using Brainware software (TDT). Recordings continued for a period of between 8 and 25 hours, until a deterioration in the physiological condition of the animal, typically either edema of the auditory cortex or cardiac arrhythmia necessitated termination. Physiological measures of auditory responsiveness (thresholds, sharpness of tuning) were monitored both on and off-line. These were comparable in all four animals and showed no deterioration over the course of the experiments.

### Stimuli

**Fig 1:**
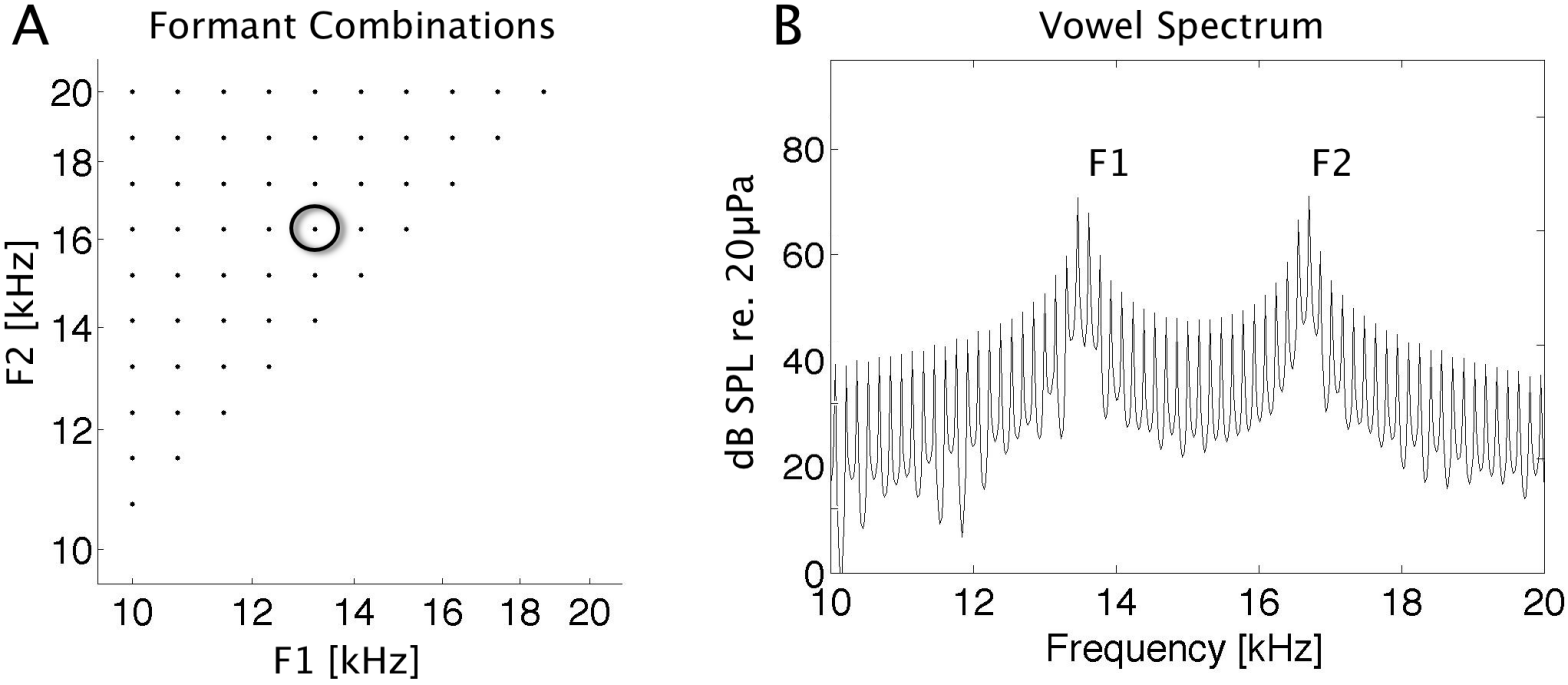
Formant Centers of Two Formant Pulse Resonance Sounds and Example Spectrum. A: Distribution of the 55 formant frequencies used to map out formant receptive fields. Formants are spaced between 10 and 20 kHz at 0.1 octave intervals. B: Amplitude spectrum of the example stimulus marked with a black circle in A.

Two-formant pulse-resonance sound stimuli of 200 ms length were generated in Matlab, using code adapted from the makeVowel() function of Malcolm Slaney’s Auditory Toolbox (http://cobweb.ecn.purdue.edu/malcolm/interval/1998-010/). Click trains with a fundamental frequency of 151 Hz at a sampling rate of 97656 kHz were passed through a cascade of two "formant filters" with center frequencies chosen at two of 11 positions between 10 kHz and 20 kHz logarithmically spaced at 0.1 octave intervals. This resulted in a set of 55 two-formant combinations. Positions of formant centers are shown in Fig 1A along with the amplitude spectrum of an example stimulus (B). 5 ms cosine onset and offset ramps were applied to the stimuli, and their amplitudes were scaled to achieve the desired RMS sound level of 75 dB SPL. The digital stimulus waveforms were converted to analog with an RX8 (TDT) digital signal processor and played through a Visaton (Haan, Germany) FRS 5 free field loud speaker. Before the start of the experiment the loud-speaker's impulse response was measured using Golay codes (Zhou et al., 1992) and a Bruel & Kjaer condenser microphone (model 4165) and measuring amplifier, and digital inverse filters were constructed to flatten the transducer output to +/- 3dB over a range of 1–35 kHz. Stimulus sound levels were calibrated to 75 dB SPL irrespective of formant frequency, using the Bruel & Kjaer microphone and measuring amplifier.

### Data Analysis

#### Spike Detection and Construction of FRFs

Offline analysis of the data was performed in Matlab (The MathWorks, Inc., Natick, MA, USA). Neural signals were bandpass filtered between 200 Hz and 2000 Hz for spike detection. Spikes were detected in each extracellular voltage trace by finding all negative peaks which exceeded a threshold level of -3.5 standard deviations of the signal amplitude distribution. To map out the formant receptive field of each recording site, each of the 55 two-formant stimuli described above was presented 15–20 times in pseudorandom order with a gap of 300 ms after the end of each stimulus, yielding a total of 500 ms interval between subsequent stimulus presentations. The measure of response strength for a stimulus was chosen to be the onset response spike rate, measured in a window of 20–100 ms after stimulus onset, minus the background firing rate, which was estimated by the median spike rate over all stimuli and repeats in a “baseline” time window 400–480 ms after stimulus onset. Receptive fields for formant combinations were constructed by averaging the response strength over all trials per stimulus. To estimate the resolution at which formant receptive fields (FRF) operate each FRF was then analyzed with 2-dimensional Gaussian filtering and cross-validation method, explained based on an example FRF in the Results section.

#### Inclusion criterion

To test for significant responses, we subdivided the pulse-resonance sound stimuli into four separate, approximately equal sized "quadrants" of the F1-F2 formant space as follows: Stimuli with F2<13.2 kHz (the "bottom left portion" of the formant space shown in Fig 1A) belonged to quadrant 1, stimuli with F1 > 13.2 kHz ("top right" part of the sampled formant space) belonged to quadrant 2, stimuli with F1+F2 > 26.4 kHz ("top left") belonged to quadrant 3, and the remaining stimuli ("central" part of the formant space) belonged to quadrant 4. We then performed a Kruskal-Wallis ("non-parametric ANOVA") test comparing median spike counts in the response time windows for the stimuli of each of the four quadrants against each other and against spike counts in the "baseline" time window. Multi-units were thus deemed to exhibit “significant” (at a level of alpha = 0.05), stimulus related modulation of their firing rates if median firing rates in any of the four stimulus quadrants differed either between each other or from the baseline firing rate.

## Results

### Responses to Two-Formant Stimuli

We recorded formant receptive fields in a total of 27 penetrations in 4 animals. Each penetration was performed with a linear multi-electode array, inserted perpendicular to the cortical surface, under visual guidance, so that the most superficial electrode site came to sit just below the cortical surface. The electrode recording sites thus spanned the entire thickness of the cortex and sampled a "cortical column" at 16 depths, from the surface to a depth of 1.5 mm in 0.1 mm intervals. Twenty-four penetrations were carried out with this 16 channel linear array electrode, and a further 3 penetrations were performed with a 32 channel electrode array, which sampled cortical columns at 32 depths at 0.05 mm intervals. This yielded a data set of multi-unit activity from 480 recording sites in total. Using the inclusion criteria described in the methods, we confirmed that the overwhelming majority of recording sites in our sample (472/480 or ca 98%) exhibited statistically significant responses to the stimuli. We excluded the 8 sites which failed to show significant responses from further analysis.

**Fig 2:**
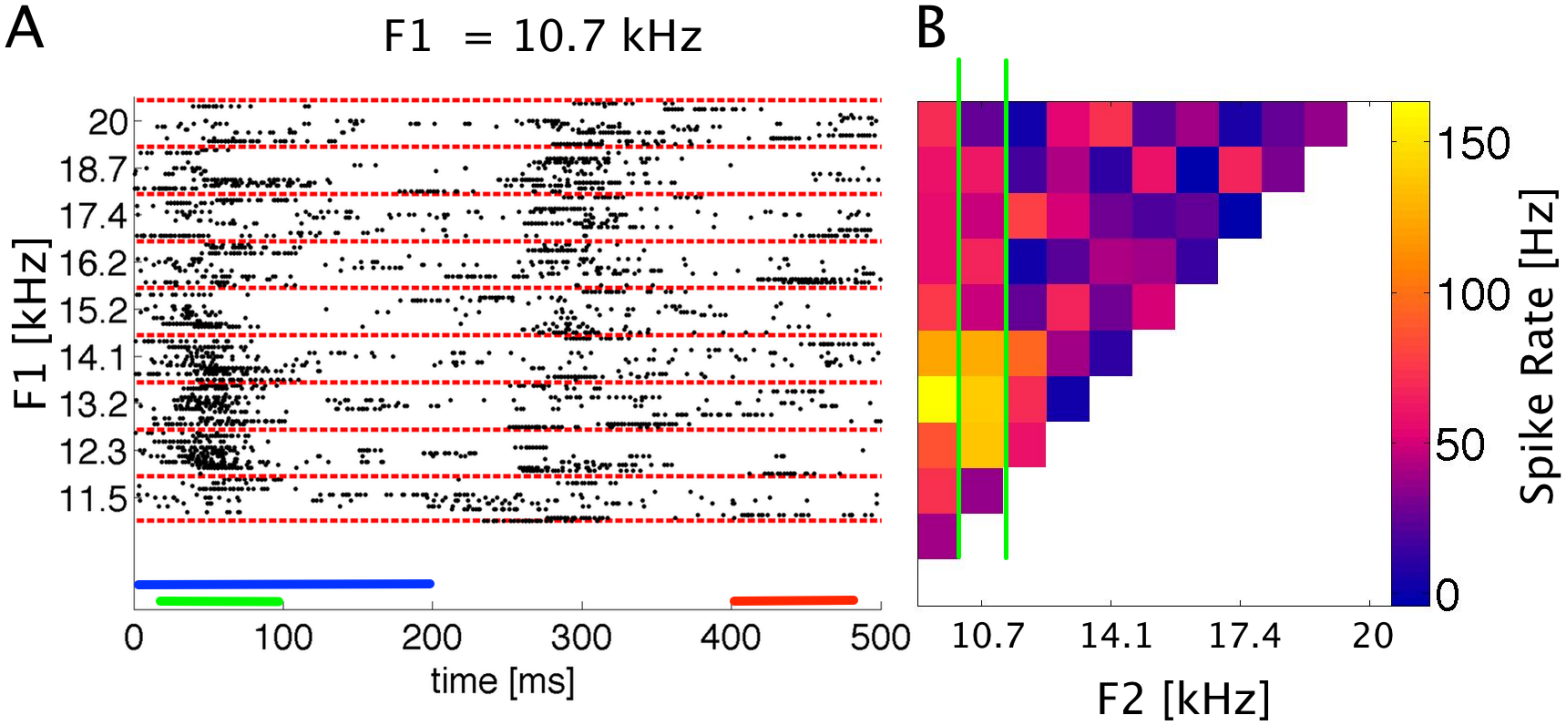
Construction of FRFs from spike times. A: Raster plot of spike responses to two formant stimuli with constant F1 of 10.7 kHz and F2 as indicated on the left. Each dot shows the timing of one action potential, each row of dots the response to one stimulus. Bars: blue = stimulus (200 ms), green = onset window (20–100 ms), red = baseline window (400–480 ms). Fifteen responses are shown for each stimulus (demarcated by the red dashed lines). B: Formant receptive field constructed by averaging spike rates in the onset response time window (20–100 ms). Color coded response values are mean spike rates in the onset window in Hertz. The column of data demarcated by the green lines (F1 = 10.7 kHz) was constructed from the raw spike data shown in A. Note that panels A and B share the same abscissa.

For each of the remaining 472 responsive recording sites we constructed formant receptive fields (FRFs), as illustrated in Fig 2. Fig 2A shows, in raster plot format, an example of spikes evoked by a subset of 9 different stimuli. The number of spikes in the evoked response varies substantially as a function of the stimulus: F2 frequencies of 12.3 and 13.2 kHz evoke onset responses which are on average two to four times as large as those seen for other values of F2. However, there is a great deal of trial-to-trial variability in the responses to repeats of identical stimuli. For example, a stimulus with F2 = 15.2 kHz sometimes evoked a strong burst of action potentials, but at other times evoked no response at all. Indeed, for this mutiunit as for many others in our data set, the spike count distributions are “Poisson-like” in the sense that the trial to trail spike count variance is of a similar magnitude to the mean spike count. A fair amount of background activity, which does not appear to be stimulus related, can also be seen in the late part of the recording window. This includes occasional “spontaneous” bursts of action potentials, and in the onset response (20–100 ms) there is no simple way to distinguish stimulus evoked activity from background activity. This level of response variability is of course quite typical for responses of sensory cortical neurons.

Fig 2B shows the formant receptive field for the same recording site, constructed by averaging the firing rates in the onset response windows for each stimulus and plotting them on a color scale against the corresponding formant frequencies. At this particular recording site, the strongest responses were seen for combinations of relatively low formant frequencies (F1 near 10 kHz, F2 near 13 kHz).

**Fig 3:**
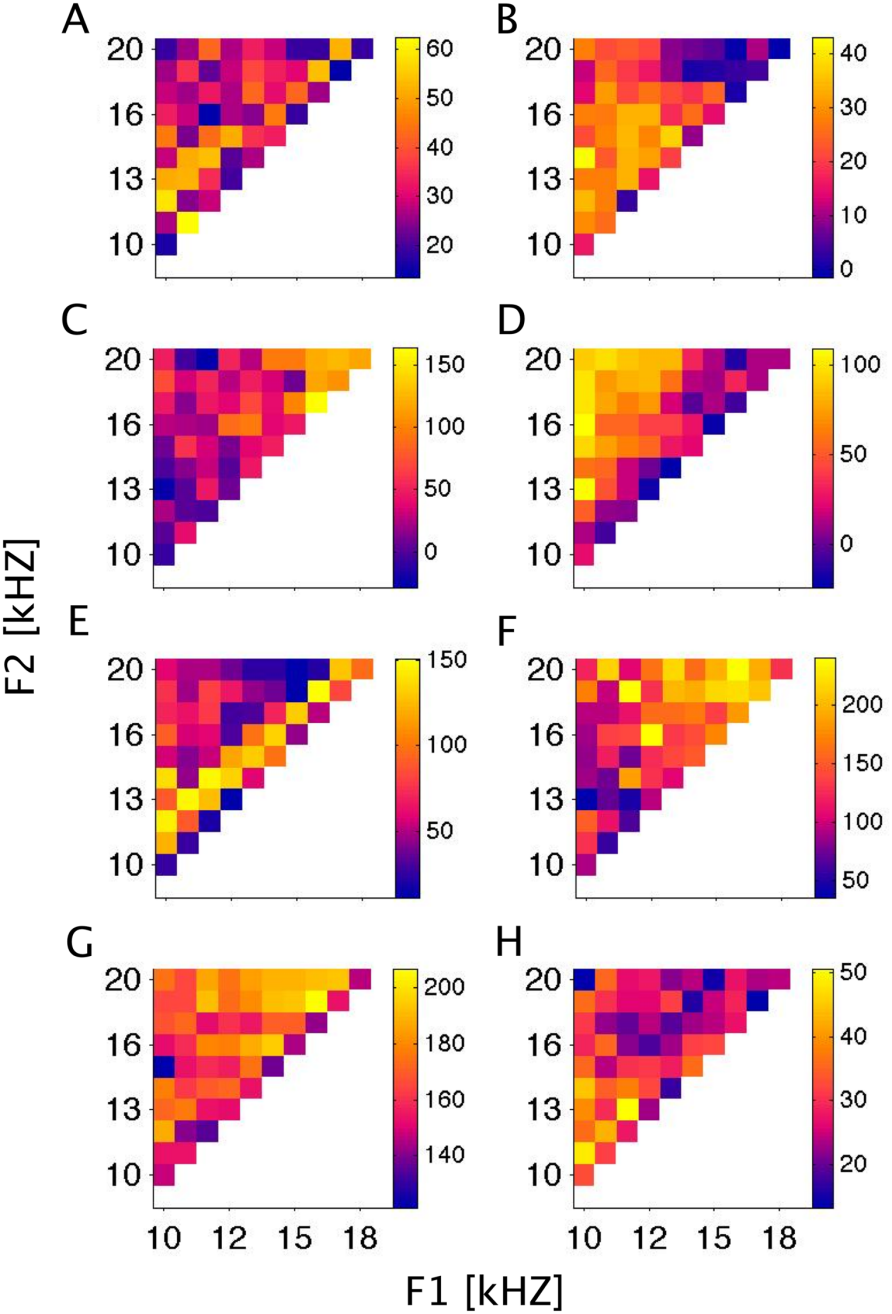
Example FRFs. Examples of formant receptive fields from a variety of recording sites, plotted as in Fig 2B. Color scales give spike rates in Hz. These FRFs are representations of mean spike rates in an onset window (20–100 ms post-stimulus) adjusted by the median spike rate in a baseline window (400–480 ms). It is not immediately clear how much of the observed structure of the FRFs is due to sampling noise, and how much reflects genuine receptive field properties.

The FRF examples in Fig 3 are chosen to illustrate the diversity of formant tuning functions we observed. Each row of FRF examples in Fig 3 comes from a different animal. The receptive field maps exhibit a variety of features: for example Fig 3B exhibits a relatively broad preference for stimuli with F1 below 15 kHz, largely irrespective of F2, while the example in 3C prefers sounds in which both F1 and F2 are relatively high. These are “broad” features or trends in the FRFs, but more fine grained features also occur: for example Fig 3E shows an intriguing and relatively narrow "diagonal stripe" which suggests that neurons at this recording site are not so much tuned to particular frequency ranges for F1 and F2, but are excited if F1 and F2 are spaced close together, within two to three tenths of an octave of each other.

Figs 2 and 3 illustrates the "variable" nature of cortical responses to the pulse-resonance stimuli. The mean responses do not drop off smoothly and monotonically as a function of distance from a best stimulus. Instead, the FRF looks "grainy". This "graininess" is likely to be a consequence of stimulus-unrelated sampling noise (Fig 2A), but some of these “fine grained features” describe the underlying neural tuning. The goal of this study is to estimate the extent to which neural responses follow changes in stimulus features (here formant center frequency). But from the average responses alone it is not clear at all which of the changes in the responses are genuine and which are merely sampling noise. So to estimate neural resolution requires us to take into account the level of sampling noise. In the following we develop and apply a method that manages to do just that.

### Estimating sensory resolution through kernel smoothing

**Fig 4:**
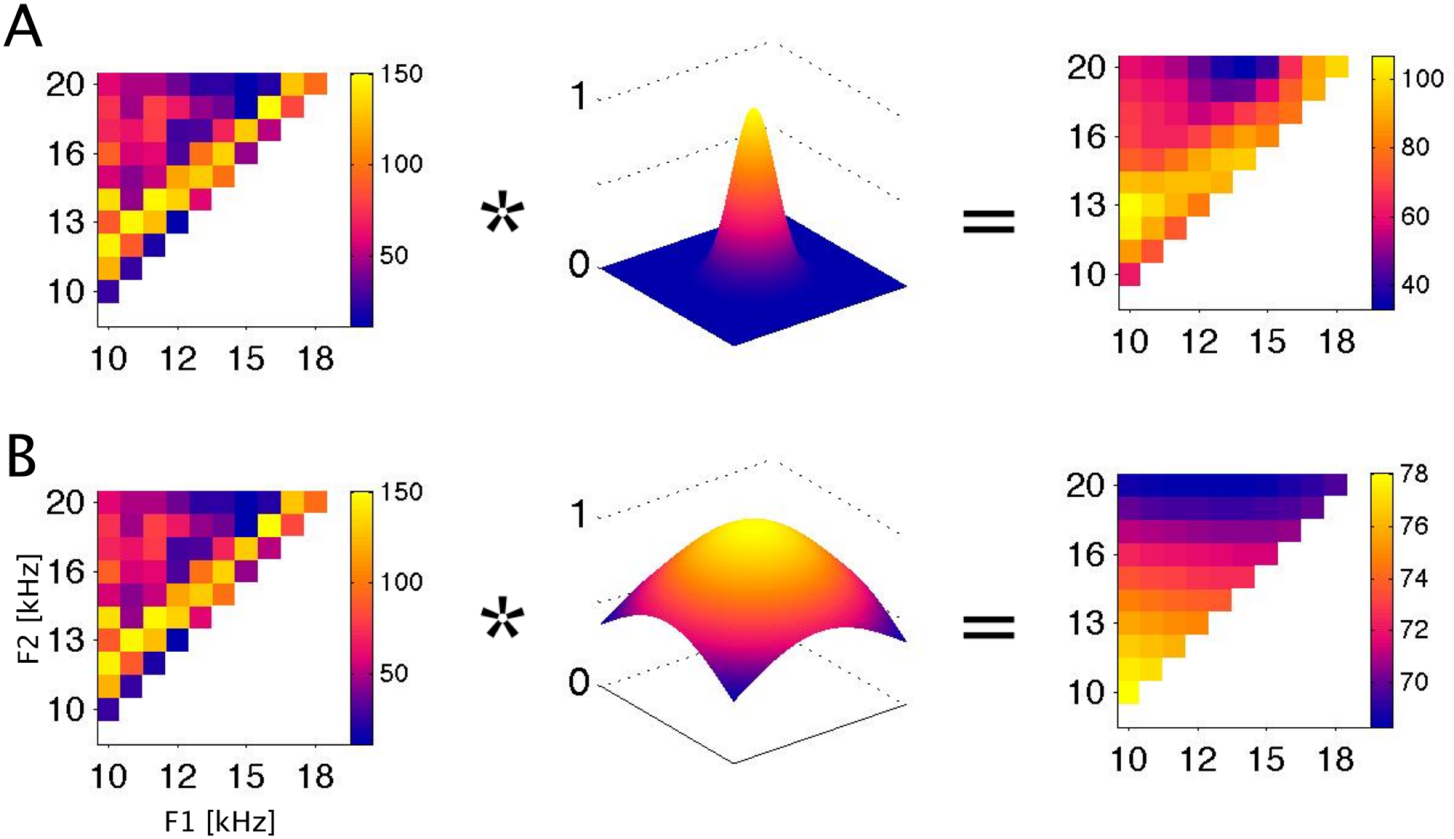
Smoothing of an FRF with 2-dimensional Gaussian smoothing filters. A: smoothing with a 0.1 octave wide filter. B: smoothing with a 0.5 octave wide filter. Left panels: The original FRF is same example as the one in Fig 3E. FRFs are plotted as in Figs 2 and 3, with formant frequencies in kHz on the coordinate axes, and the color scale giving spike rates in Hz. Middle panels: 2-dimensional Gaussian filters with σ = 0.1 (A) or σ = 0.5 (B) octaves standard deviation. Right panels: The result of convolving the FRF with the smoothing filter. To determine the ‘best’ σ for individual FRFs we use a cross-validation method described in Fig 5.

Sampling noise is usually “cleaned up” through the use of smoothing filters. Fig 4 illustrates the effect of smoothing the FRF from Fig 3E by convolution with a 2-dimensional Gaussian filter (or “kernel”) with a width (standard deviation) of either 0.1 or 0.5 octaves.

The smoothed FRFs certainly “look less noisy” and may seem easier to interpret in terms of systematic stimulus-response relationships. But the process of smoothing moves us away from the actual, “raw” experimental data, which raises the question whether the smoothed FRF is a more or a less accurate estimate of the “true” underlying neural tuning function. As it turns out, a smoothed FRF can be a more accurate representation of the underlying physiology, but only if the smoothing filter is chosen appropriately. Furthermore, determining the appropriate smoothing filter width is itself revealing about the true physiological resolving power of the neurons.

To chose a filter that accounts for the response variability illustrated in Fig 2, we can conceptualize an observed response ř to a given stimulus S as a random variable Φ, which is itself dependent on some deterministic underlying neural tuning function r, i.e. ř(S) = Φ(r(S)). One popular way to model this is the so-called linear-nonlinear-Poisson (LN-P) model, which seeks to capture the deterministic part r(S) as a linear transformation of the stimulus S followed by an output non-linearity, and the non-deterministic part Φ with a Poisson random number generator [23,24]. Of course LN-P and other, similar models are only an approximation of the physiology, and rest on a number of simplifying assumptions. However, for our analysis, the precise form of the noise process Φ and of the deterministic neural tuning function rare unimportant.

It is common practice to use the averaged observed responses ř(S) as an estimate of the “true” underlying tuning function r(S), and repetition and averaging are used to minimize the influence of the noise process Φ. Such repetition is useful because, according to the central limit theorem of statistics, the expected error in the estimate declines with the square root of the number of repeats that we have available to average over. Additional smoothing in the manner illustrated in Fig 4 may improve the estimate further because it effectively includes responses to presentations of a set of “neighboring” stimuli ř(S') into the averaging process (possibly with some weighting). This increases the effective number of observations that contribute to each data point and thereby reduces “noise”, but it genuinely improves the final estimate only if S and S' are “closely neighboring stimuli” in the sense that they can be expected to produce very similar responses. If that was not the case, the smoothing would “flatten out” genuine differences in stimulus evoked firing more than it would suppress physiological noise in the responses. In other words, smoothing relies on the fact that, to within expected measurement error, r(S)≈r(S') provided that the distance between the stimuli |S-S'| in the relevant parameter space is “small enough”, smaller than some appropriately chosen smoothing parameter σ. In practice this is usually implemented with a nearest neighbor weighting filter which weights the contributions of stimuli S' heavily if |S-S'|< σ, but the weight of these contributions should decline sharply if |S-S'|> σ. The precise shape of this weighting function is not critical. Most functions which heavily discount stimuli that are "far away" (|S-S'|>σ) are suitable and will generally yield very similar results. Rectangular smoothing windows are popular choices, as are the Gaussian kernels which we have chosen here. For instance, when smoothing with a σ = 0.1 octave wide Gaussian filter (as depicted in Fig 4A), the responses within +/- 0.1 octave from each stimulus will contribute ca 40% of the weighted average, while those within +/- 0.2 octaves will contribute 98%, so that those further than 0.2 octaves away make only a negligible contribution. Choosing an appropriate value for the width parameter σ, however, is all important. If it is too small, then the estimated neural tuning function will be noisier than it need be, but if it is too large, then excessive smoothing will blur details of the “true” FRF in our final estimate. Most commonly, previous authors have simply used their judgment to pick apparently reasonable but ultimately arbitrary smoothing parameter values [25–27]. A better, but still little known, alternative is to use cross-validation methods to optimize the choice of σ for a particular data set [21]. Estimating the optimal σ in this manner is useful not only because it produces better receptive field estimates, it is also informative because the optimized value of σ provides a direct estimate of the “stimulus resolving power” of the neural responses under study. For an optimally chosen value of σ, r(S)≈r(S') will hold for all (S, S') such that |S-S'|<σ, but not necessarily for |S-S'|>σ. This is just a more formal way of stating that two stimuli must differ by more than σ for the responses to these stimuli to be reliably different given the expected trial-to-trial response variability. In this sense, σ can serve as a measure of the “stimulus resolution limit” afforded by the neural responses.

**Fig 5:**
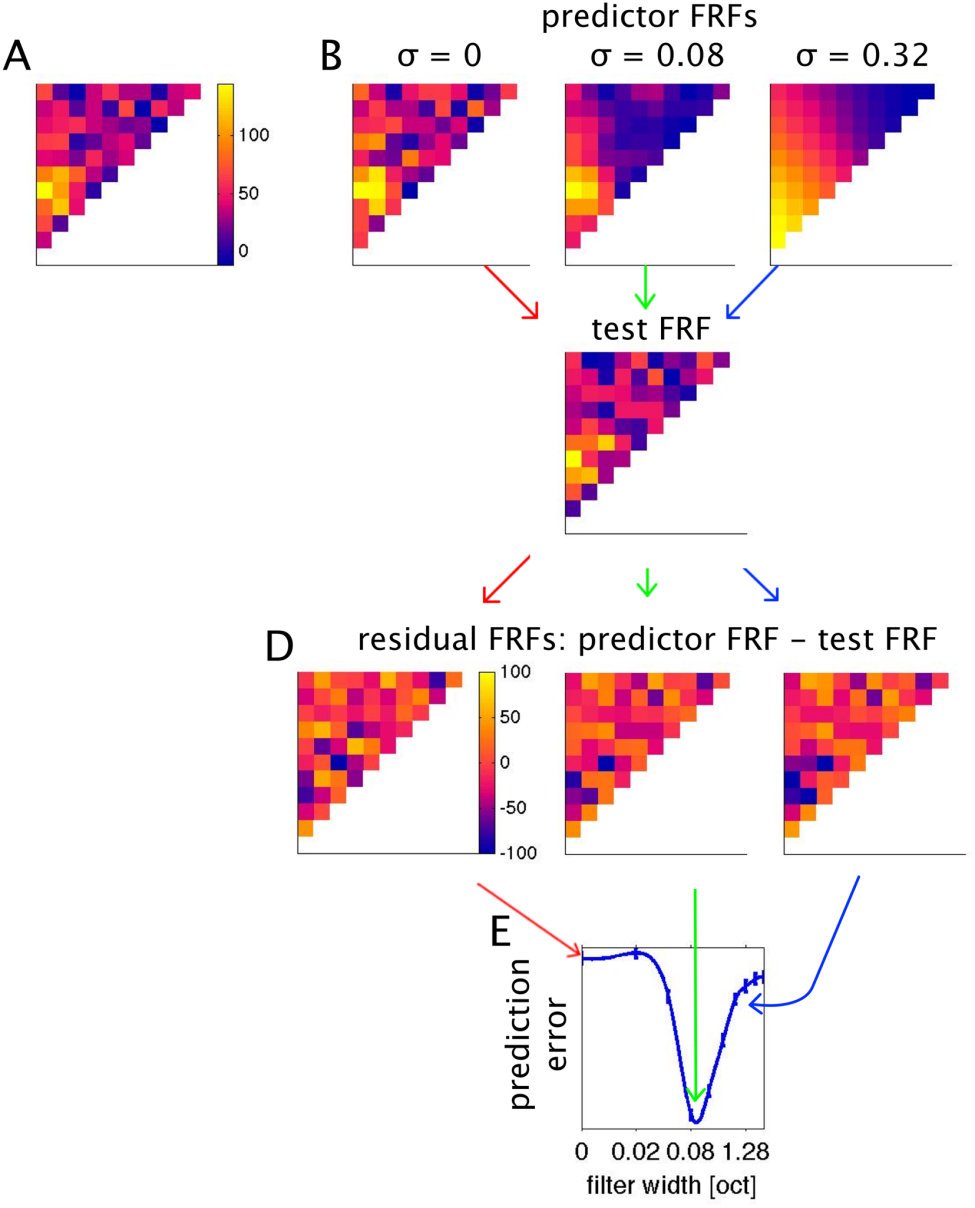
Using cross-validation to estimate optimal FRF smoothing filters. A: The original raw FRF, estimated by averaging over 15 repeats. The data set is randomly split into subsets, a “training” set, shown in B, comprising 10 repeats for each stimulus, which are smoothed with a variety of smoothing filters of varying width, and a test set, shown in C, comprising the remaining 5 repeats. D: The differences between the smoothed training sets and the test set (residuals) are calculated. E: Mean squared residual values serve as a measure of the prediction error associated with each σ. Mean and standard error of prediction errors were estimated using “bootstrap” repeated random subsampling of the data as shown in A to D for 500 iterations. Mean and SEM prediction errors are plotted connected by a spline interpolation between the sampled values. The minimum of the interpolated curve serves as the estimated optimal σ in all subsequent analysis.

The process of cross-validation used to determine the optimal value of σ (adapted from Hastie et al. 2009) is illustrated in Fig 5. The data set is repeatedly randomly split into two subsets, usually referred to as “training sets” and “test sets”. The principle is to smooth the training sets with a range of varying width parameters σ, and to find the value of σ that offers the best compromise between insufficient and excessive smoothing through trial and error by determining which smoothed training sets most accurately predicts the test sets. The number of samples used to construct the training and test sets is not critical. In the results shown here, our total data set typically comprised 15 presentations for each stimulus, and the training and test sets were made up of random subsamples (without replacement) of 10 and 5 repeats each, but a 14:1 split (“leave-one-out cross-validation”) gave essentially the same results. Fig 5B shows the effect of smoothing at varying values of σ. The mean squared difference between the “predictor FRF” (the smoothed training set) and the test set FRF serves as a measure of the prediction error. The process of randomly splitting the data and calculating prediction errors was repeated 500 times for each value of σ to generate a bootstrap estimate of the prediction errors. In this manner we calculated prediction errors for σ ∈ {0.02, 0.04, 0.08, 0.16, 0.32, 0.64, 1.28, 2.56, 5.12} octaves, and interpolated between these values with cubic splines. The value of σ associated with the lowest prediction error optimally smoothes the FRF data, and at the same time provides an estimate of the “formant frequency resolution limit” afforded by the neural responses.

**Fig 6:**
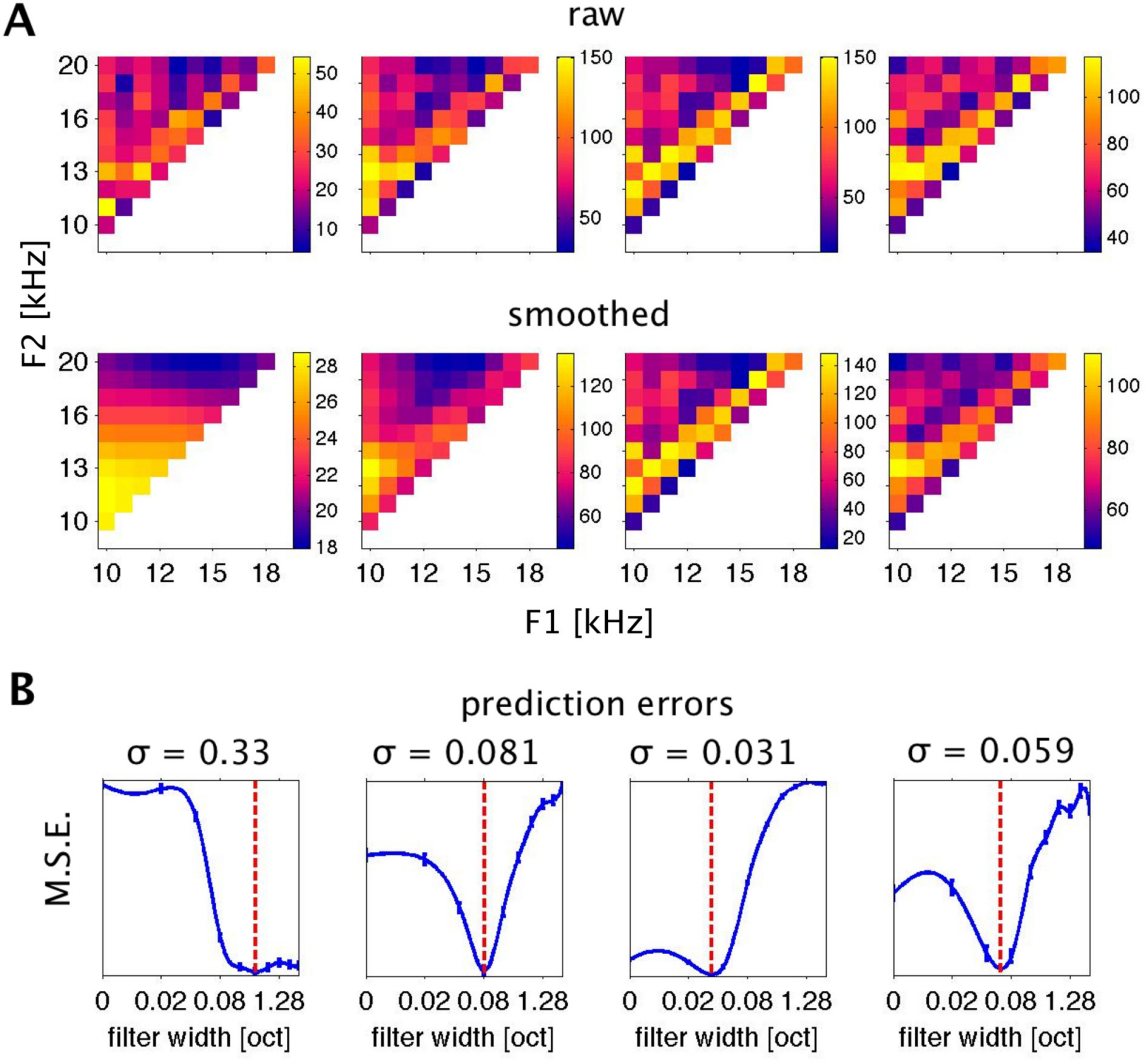
Effects of Smoothing and Determination of Optimal Filter Widths. A: Four example multiunit FRFs, without smoothing (top row) and after optimal smoothing (below). B: Bootstrap estimates of prediction errors associated with various levels of smoothing for the FRFs shown in A. Error bars are s.e.m. at the respective σ. Blue lines are at values interpolated (using a spline interpolation) between the sampled σ values. Red lines show the minima of the interpolated curves that were chosen as estimators for optimal smoothing and formant resolution and are summarized in Fig 7.

When applying this kernel smoothing analysis to a large sample of FRFs, one observes that the formant frequency resolution afforded by the neural responses differs substantially between recording sites. This is illustrated in Fig 6. The top row of Fig 6A shows examples of four raw FRFs, and just below them, the FRFs are shown after smoothing with Gaussian kernels of optimal σ. Fig 6B shows the prediction error (the mean squared error M.S.E.) associated with differing σ. The value of σ associated with the lowest prediction error is indicated above each plot.

The FRF plotted in the leftmost panels has the poorest underlying formant frequency resolution (best σ = 0.33 octaves) among the sample shown in Fig 6. Smoothing with kernels as wide as a fifth of an octave or more substantially increases the “predictive power” of the FRF, and prediction errors do not increase substantially when the kernel widths are increased further to very large values of an octave or more. This indicates that, for this multi-unit, trial-to-trial variability of neural responses is very large and stimulus related changes in response rate are comparatively small, so that the grand average response over all stimuli (produced by filters with very large σ) provides about as good a prediction for the response to any particular stimulus as one is going to get. The multi-unit's responses are therefore unable to support reliable discrimination between different formant frequencies, and the fine grained details in the raw FRF are predominantly attributable to sampling noise.

Contrast this with the multi-unit shown in the third column in Fig 6. The initial decrease of residuals is very small, indicating that smoothing the raw FRF leads to only very modest increases in predictive power, while smoothing with σ greater than a very modest 0.03 octaves substantially and significantly impairs the predictive power of the smoothed FRF. The cross validation analysis thus indicates that the complex, fine grained structure of this FRF is a “real” feature of the underlying neural tuning function, and this multiunit therefore “resolves” formant frequency steps which are only a small fraction of an octave wide because such small changes in formant frequency can lead to reliable changes in the evoked firing rate.

Before we move on to consider the statistical and anatomical distribution of σ resolution values observed across our data set, it is worth pausing to elaborate on how σ values should be interpreted. For example, the reader might wonder whether it is sensible for us to even consider σ values smaller than the stimulus step size of 0.1 octaves used during the data collection. Does the inherent resolution limit of 0.1 octaves in our experiment not preclude the possibility of investigating σ resolution values smaller than 0.1 octaves? When interpreting σ values it is important to bear in mind that these are “standard deviations” of a Gaussian filter function, and stimuli as far as ±2σ or further away from the filter's center frequency will make non-negligible contributions to the filter output. For example, when we set σ = 0.08, responses to stimuli 0.1 octaves removed from the center of the filter will contribute to the spatially averaged output with a sizable weight, corresponding to 45% of the weight of the response at the center of the smoothing filter. For σ = 0.04, the weighting of stimuli 0.1 octaves away from the center drops to a modest 4.4%, and at σ = 0.02 it becomes a completely negligible 3.7 parts per million. Bearing this in mind, if we analyze an FRF with Gaussian filters as illustrated in Fig 6 and find the optimal value of σ to be 0.04 octaves but no smaller, then that indicates that the smoothing afforded by adding 4.4% weighted contributions from the responses to each of the 0.1 octave distant “nearest neighbor” stimuli tested to the weighted average response improves the predictive power of the smoothed FRF in the cross-validation test. Thus, an “optimal σ” of 0.04 therefore does not imply that a multiunit can necessarily reliably signal a 0.04 octave change in formants. It merely implies that the influence of stimuli 0.1 octaves away is small but greater than zero, otherwise factoring in responses to those stimuli in the smoothed FRF would lead to a degradation, rather than an improvement, of the FRF's power to predict the responses in the cross-validation set. The contribution made by stimuli away from the center of the filter falls to less than 1% at ~3σ, and rapidly asymptotes to zero for increasing σ. It is therefore reasonable to interpret a particular value of σ as evidence that responses to frequencies more than 3σ apart are independent, but frequencies less than 2σ apart are not. Furthermore, if we were to find the best σ for a particular multiunit to be 0.02, the smallest value tested, then that would imply that the multiunit's “frequency resolving power” is too small to measure with the 0.1 octave spaced stimulus set used in this experiment. As we will see, none of the multiunits in our data set had optimal values of σ below 0.04.

### Neural Resolution of Formant Frequencies

**Fig 7:**
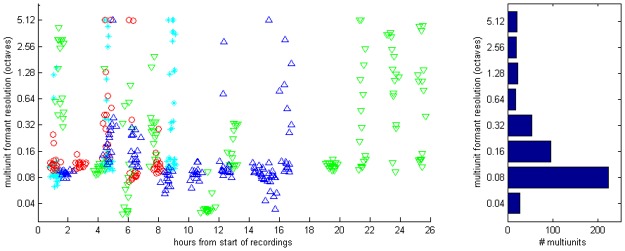
Distribution of best filter width, as estimated with the kernel smoothing and cross-validation method. A: observed multiunit resolutions plotted as a function of time since the start of each recording experiment. Each symbol shows the formant resolution of one multiunit. Different symbols and colors are used to distinguish data from different animals. A random Gaussian jitter with a standard deviation of 0.2 has been added to the time coordinate to spread out overlapping data points from simultaneously recorded multiunits. B: Distribution of filter widths summarized in histogram form. The median filter width lies at 0.1161.

**Fig 8:**
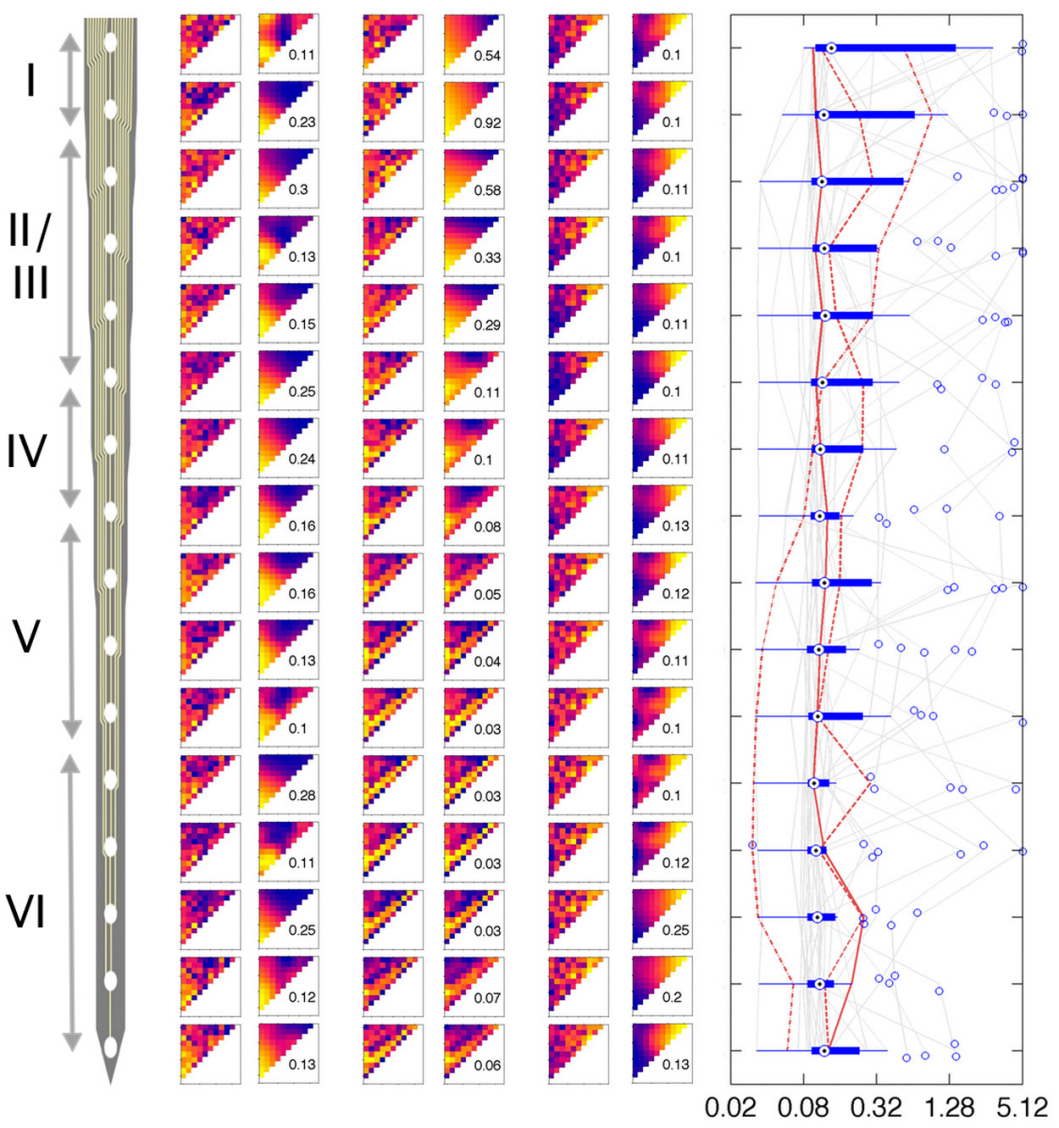
Development of filter width with cortical depth. Left is a schematic of the 16-channel probe used to perpendicularly penetrate rat auditory cortex. Channel 1 represents the pial surface and channels are 100 microns apart. In the middle panel FRFs from 3 animals (left columns: raw data, right columns: smoothed at optimal σ) at each of the 16 channels are shown. The right panel shows box plots of optimal σ at the respective cortical depth over all 27 penetrations (total 472 data points).

**Fig 9:**
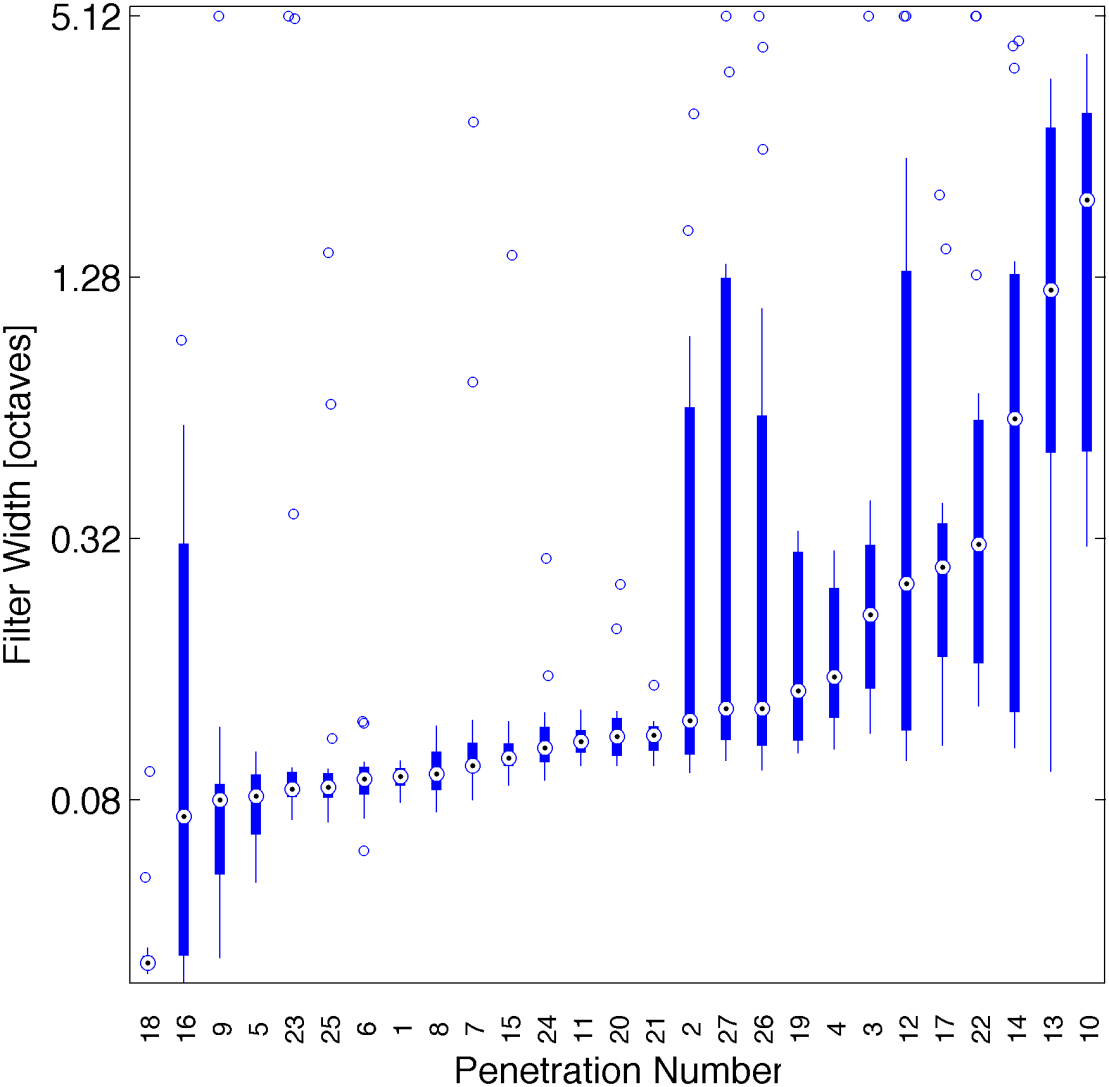
Difference of filter width with cortical location. The box plot shows the distribution of best σ values sorted by median for each array electrode penetration. Penetrations 1–9, 10–18, 19–24 and 25–27 are from different animals. Median σ values differ highly significantly from one penetration to the next in all 4 animals (Kruskal-Wallis Test: p << 10^−12^).

The substantial differences in formant frequency resolution which are illustrated in Fig 6 raise the question: how is formant frequency resolution distributed across the cortical population, both statistically and anatomically? Are some formant frequency resolutions found more frequently than others? Do highly or poorly resolving multi-units tend to cluster in cortical layers or cortical columns? Figs 7–9 address these questions. Fig 7 shows the distribution of best filter widths for all 472 multi-units which responded to the pulse-resonance sounds. The panel on the left shows them as a scatter plot against time after the start of the electrophysiological recordings, with data from each animal plotted with a different symbol. Plotting the data in this manner visualizes the distribution obtained from each multielectrode penetration, shows that the distributions were similar in each of the four animals, and that there were no obvious trends for the quality of the data deteriorating during the course of the experiment. The panel to the left summarizes the observed formant resolution values in histogram form (it shows the “marginal” distribution of the left panel). While many multielectrode penetrations exhibit a wide spread of formant frequency resolutions, resolutions of around one tenth of an octave are clearly the most numerous. Approximately two thirds of the multi-units have a formant frequency resolution between 0.05 and 0.2 octaves. The median best filter width over all 472 multi-units is 0.1116 octaves. This corresponds to a shift in formant frequency of approximately 8%.

We observed no obvious relationship between optimal filter width and the median response strength of multi-units (data not shown), and even relatively weakly responding multi-units sometimes exhibited the reliable dependence of firing on stimulus parameters that is necessary for FRFs which resolve formant frequencies with high acuity.

We also examined whether cortical depth or location (or both) had a systematic influence on formant frequency resolution of two-formant PRSs. Fig 8 shows examples of FRFs recorded from three different “cortical columns” (i.e. three penetrations with electrode arrays orthogonal to the cortical surface which record neural activity at regular 100 μm intervals). Both raw and optimally smoothed FRFs are shown. The examples illustrate that FRFs recorded at one particular depth often, but not always, closely resemble those recorded in the sites immediately above or below. Thus there appears to be a clustering of formant tuning properties within cortical columns which is somewhat reminiscent of “orientation tuning columns” described in primary visual cortex [28], even if a particular shape of FRF is not usually sustained through the entire depth of the cortical column. Also, formant frequency resolution (best σ) can vary considerably as a function of cortical depth, but there is no clear systematic relationship between formant resolution and cortical depth. The right panel of Fig 8 shows the distribution of formant resolution as a function of cortical depth. Data at odd numbered electrode sites from the three 50 μm spaced 32 channel electrode penetrations were omitted here for clarity to yield a sample of 432 sites sampled at 100 μm intervals in 27 penetrations. The anatomical distribution of optimal σ along the three example penetrations is highlighted by the three red traces in the same panel. While some penetrations may exhibit a wider spread of formant resolution values throughout the depth of penetration, this appears not to be the general case, and there is no very clear dependence of formant resolution on cortical depth. Median resolution values are similar at all depths in most penetrations, and they exhibit no statistically significant dependence on depth (Kruskal-Wallis test, p>0.2).

But while cortical resolution of formant frequencies did not depend systematically on depth, there were substantial and statistically highly significant variations from one penetration to the next. In other words, while some cortical columns appear to resolve formant frequencies in fine detail, others do not. This is illustrated in Fig 9, which shows the distribution of optimal σ for each of the 27 penetrations, sorted by the median optimal σ. The apparent, large frequency resolution differences from one column to another were statistically highly significant (Kruskal-Wallis Test: p << 10^−12^).

## Discussion

In this study we examined the tuning of neural responses in A1 of rats to formant frequencies of two-formant pulse resonance sounds. The stimuli used here provide a simple, experimentally tractable approximation of the types of pulse-resonance sounds that are widely used in human and animal communication. That neurons in A1 are clearly very sensitive to these types of sounds can be inferred from the fact that we observed statistically significant responses at 98% of recording sites in our sample. The structure of the observed FRFs was, however, highly diverse, and the fact that many neurons exhibit large trial to trial response variability makes it difficult to distinguish “real” detail in the FRFs from sampling noise in the raw data. To deal with this diversity and variability we deployed a kernel-smoothing method [21], which uses cross validation to discover an optimal neighbor weighting function (or "kernel") which maximizes the predictive power of the smoothed receptive field data. This allowed us to optimize our FRF estimates and at the same time quantify the formant frequency resolution afforded by the neural responses.

### Cortical Resolution of Formant Combinations

We found that the majority of best smoothing filters had a width of less than 0.2 octaves, with a median best σ of 0.11 octaves. The method we used to generate these estimates of the “neural resolution” has, to our knowledge, not been previously used in sensory neurophysiology, and some comments on how these σ values should be interpreted and compared to results obtained from previous studies with very different methodologies are clearly called for. A variety of methods used to quantify neural tuning bandwidths is in use in the auditory literature, each incorporating different conventions, which make exact comparison of the values obtained with the various method difficult, and one might wonder whether the new method we introduce here does not just add to the confusion. However, firstly, as we will discuss below, our method has unique strengths which make it particularly suitable for certain types of study, and secondly, while an exact comparison of different types of neural or psychoacoustic measures of bandwidth or frequency resolution is difficult, one can nevertheless discern quite easily whether the different methods give results which are in approximate, qualitative agreement.

For example, Barbary ([29] reports Q20 factors for rat auditory nerve fiber tuning at characteristic frequencies near 10 kHz to be about 4, implying a bandwidth of 10/4 = 2.5 kHz. Meanwhile, Ruggero & Temchin [30] argue that Q10 values are more readily comparable to psychoacoustically determined measures of frequency tuning, and calculate these to be about 6 for rats at 10 kHz, corresponding to a bandwidth of 10/6 = 1.66 kHz. How does that compare to the median best σ values of ca. 0.11 octaves we observed? As already mentioned in the results, these values specify the standard deviations of a 2-D Gaussian filter, and as such are possibly better through of as an estimate of the “half-bandwidth”, rather than the bandwidth, of the underlying “sensory filters”. If we consider values of 2σ as approximate “cortical bandwidth” estimates, then our median “cortical bandwidth” in the rat would be approximately 0.22 octaves, corresponding to ca 1.6 kHz bandwidth at a 10 kHz center frequency. The “cortical formant resolution”we have observed is therefore quite similar, and apparently no larger, than auditory nerve fiber frequency resolution reported in the literature, which suggests that A1 makes efficient use of the available cochlear frequency resolution in its representation of formant frequencies of vocal sounds.

Comparing the values we obtained against psychoacoustic thresholds might be similarly interesting. Sadly there are no behavioral studies of formant discrimination thresholds in rats, but pure tone discrimination thresholds have been published by Syka et al. [31], who report Weber ratios of 5.7% on average, and by Talwar and Gerstein [32], who observed similar, if slightly better, Weber ratios in the range of 3.06 ± 0.44%. In comparison, the auditory nerve fiber and cortical formant resolution “bandwidths” just discussed, in the order of about 16%, appear rather broad. If we assume that behavioral formant discrimination thresholds are similar to those seen in pure tones, and that these behavioral thresholds need to be achieved from a readout of cortical responses, then it appears that this readout may need to achieve a degree of “hyper-resolution” to reach observed psychoacoustic thresholds.

Of course, how such a readout would operate, and how much any one cortical neuron or multiunit would contribute to it, is still largely unknown, although Bizley and colleagues [33] have demonstrated that even relatively naïve vector decoding of responses from small ensembles of auditory cortical neurons can be sufficient to substantially improve the tuning of a neural ensemble to the fundamental frequency of an artificial vowel relative to the tuning of the individual neurons which constitute that ensemble, and that the improvements achieved by reading responses from modest sized ensembles of less than 20 neurons are often sufficient to account for behavioral thresholds.

Thus, achieving highly accurate readouts is not difficult if ensembles are large and the activity of individual responses of the neurons in the ensemble is “informative”, but the readout accuracy is limited by the degree of redundancy in the information inherent in the tuning of individual neurons [34,35] as well as by “noise correlations” in the firing of the neural ensemble [36] which would further reduce the amount of independent information which each neuron can contribute. While the neural resolution measure we developed here is sensitive to the noise inherent in trial-to-trial variability of discharges, it contributes nothing to our understanding of such informational redundancy in neural population codes. Furthermore, there are other measures which are in widespread use in central auditory physiology which quantify neural coding in the light of response variability, such as Shannon information [6,17,37,38] or Fisher information [39,40]. So why, or when, is the use of a neural resolution estimate through kernel-smoothing of the type developed here more appropriate than the use of alternative, already well established methods for the quantification of neural tuning or coding? Information-theoretic measures are by design agnostic about the value or significance of individual “signals” and merely quantify their average discriminability. In contrast, in the perceptual interpretation of sound signals, some discriminable differences between signals may be highly relevant, and others not, and we might expect the shape of the tuning function to reflect which distinctions “matter” to the neurons. The fact that our kernel-smoothing method, rather than disregarding the shape of the underlying neural tuning function, instead produces an optimized estimate of it, can therefore be advantageous. To give a specific example, consider the FRF shown in Fig 3D, which features a prominent “diagonal”, which indicates that this multiunit may have a preference for pulse-resonance sounds whose formants have a fixed relationship to one-another, but irrespective of the precise value of the lower of the two formants. Such tuning properties would be compatible with the intriguing hypothesis put forward by Patterson and others [41] that the higher levels of the auditory system construct a “scale invariant” representation of vocal communication sounds which normalizes out the length of the vocal tract of the vocalizing animal. Or alternatively, the diagonal feature could just be noise artefact. Given that we found the formant resolution value σ for this particular multiunit to be as small as 0.031 octaves (compare Fig 6), that banal explanation can be discounted. The diagonal feature in this particular example is “robust”, as well as intriguing, all the more so since this multiunit shares that diagonal feature with neighboring multiunits in the deeper layers of the same cortical column (compare Fig 8). It is tempting to interpret this example as preliminary evidence for the emergence of scale-invariant representations of formant structure in the deeper layers of auditory cortex, but one must of course be must be careful not to read too much into a small number of examples, and a comprehensive assessment of the likely functional significance of FRF shapes will have to await future work with substantially larger data sets. But in any event, kernel smoothing of the observed receptive fields is bound to provide a valuable, if not essential, pre-processing step.

### Anatomical Distribution of Cortical Formant Resolution Values

Our investigation of the anatomical distribution of formant tuning parameters led to three main observations. First, there is no consistent depth profile for formant resolution across our penetrations. Second, while changes across cortical layers revealed no significant canonical pattern, there may be a trend towards more homogeneous resolutions in layers 5/6. And third, overall formant resolution varies highly significantly from one penetration to the next, in other words there appear to be “patches” of sharper and broader formant frequency tuning.

Within each penetration we found that formant resolution (and indeed the FRF structure) is homogeneous along neighboring recording sites. There is no obvious systematic depth profile across penetrations, and roughly two thirds of all recorded sites give σ values between 0.05 and 0.2 octaves (median ~0.11). Similar trends for tuning properties to remain similar across depth for individual penetrations normal to the surface of auditory cortex have been observed for pure tones [42,43], intensity tuning [44], frequency sweep tuning [45] as well as binaural integration properties [46,47], and these observations have often been interpreted as evidence for a "columnar" functional organization of auditory cortex.

However the picture of essentially homogeneous tuning to a particular set of acoustic features throughout different lamina of A1 begs the question what computations such columnar networks of cortical cells contribute to the processing of the stimuli, as one might “columnar information processing” to induce systematic changes in the tuning to acoustic features as information passes from thalamorecipient, granular layers to supra- and then infragranular layers. In the primary visual cortex, such systematic changes have been described for receptive field size and sensitivity to spatial phase [48]. In contrast, in auditory cortex, the evidence for columnar transformation of acoustic information has been described as “contradictory and inconclusive” [49] and until recently the only simple neural response features which were known to differ systematically across layers were response latency [50,51] and perhaps also stimulus specific adaptation [52].

Our results similarly reveal no widespread, systematic dependence of formant resolution on depth. Fig 8 suggests a trend that formant tuning properties may be more heterogenous in the superficial layer, which would be consistent with very recent investigations of pure tone tuning properties in the auditory cortex of the mouse [53]. However, while we found little evidence for any systematic depth dependence, individual penetrations appearently can have particular depth profiles. This observation is supported by the highly significant differences in median resolution between penetrations illustrated in Fig 9. This is indicative of a patchy organization of cortex, where each cortical column may be processing sounds in a slightly different manner, and while many cortical columns are quite sharply tuned for formants, others are not. Such a patchwork-like organization is in agreement with recent reports that units at different cortical locations integrate the features of vowel-like sounds in a very diverse manner [4,6].

In conclusion, the evidence for information processing within columns of auditory cortex remains largely inconclusive today, 57 years after Mountcastle coined the term “cortical column” to describe his observations in cat V1. Our results similarly indicate a distributed, patchwork-like anatomical organization of formant frequency resolution in auditory cortex which is diverse both within and across cortical columns. Yet, while cortical locations vary widely in their resolution of formant frequencies, most exhibit resolutions of around 0.11 octaves, a value which is broadly comparable with behaviorally determined pure tone discrimination thresholds in rats.

## References

[1] Patterson RD, Smith D, van Dinther R (2007) Size information in the production and perception of communication sounds Auditory Perception of Sound Sources. Chapter 3. p. 43–75, Springer Handbook of Auditory Research, Springer, Boston, MA

[2] Schnupp J, Nelken I, King A (2011) Auditory Neuroscience. MIT Press 1 pp.

[3] Ohl FW, Scheich H (1997) Orderly cortical representation of vowels based on formant interaction. Proc Natl Acad Sci USA 94: 9440–9444. 9256501 10.1073/pnas.94.17.9440 PMC23209

[4] Bizley JK, Walker KMM, Silverman BW, King AJ, Schnupp JWH (2009) Interdependent encoding of pitch, timbre, and spatial location in auditory cortex. Journal of Neuroscience 29: 2064–2075. 10.1523/JNEUROSCI.4755-08.2009 19228960 PMC2663390

[5] Mesgarani N, David SV, Fritz JB, Shamma SA (2008) Phoneme representation and classification in primary auditory cortex. J Acoust Soc Am 123: 899–909. 10.1121/1.2816572 18247893

[6] Walker KMM, Bizley JK, King AJ, Schnupp JWH (2011) Multiplexed and robust representations of sound features in auditory cortex. Journal of Neuroscience 31: 14565–14576. 10.1523/JNEUROSCI.2074-11.2011 21994373 PMC3272412

[7] Bendor D, Wang X (2005) The neuronal representation of pitch in primate auditory cortex. Nature 436: 1161–1165. 16121182 10.1038/nature03867 PMC1780171

[8] Grimsley JMS, Shanbhag SJ, Palmer AR, Wallace MN (2012) Processing of communication calls in Guinea pig auditory cortex. PLoS ONE 7: e51646 10.1371/journal.pone.0051646 23251604 PMC3520958

[9] Syka J, Šuta D, Popelář J (2005) Responses to species-specific vocalizations in the auditory cortex of awake and anesthetized guinea pigs. Hear Res 206: 177–184. 16081007 10.1016/j.heares.2005.01.013

[10] Obleser J, Lahiri A, Eulitz C (2003) Auditory-evoked magnetic field codes place of articulation in timing and topography around 100 milliseconds post syllable onset. Neuroimage 20: 1839–1847. 14642493 10.1016/j.neuroimage.2003.07.019

[11] Prather JF, Nowicki S, Anderson RC, Peters S, Mooney R (2009) Neural correlates of categorical perception in learned vocal communication. Nat Neurosci 12: 221–228. 10.1038/nn.2246 19136972 PMC2822723

[12] Bar Yosef O, Rotman Y, Nelken I (2002) Responses of neurons in cat primary auditory cortex to bird chirps: effects of temporal and spectral context. Journal of Neuroscience 22: 8619–8632. 12351736 10.1523/JNEUROSCI.22-19-08619.2002 PMC6757805

[13] Machens CK, Wehr MS, Zador AM (2004) Linearity of cortical receptive fields measured with natural sounds. Journal of Neuroscience 24: 1089–1100. 14762127 10.1523/JNEUROSCI.4445-03.2004 PMC6793584

[14] Linden MSJF (2003) How linear are auditory cortical responses? Advances in Neural Information Processing Systems 15: Proceedings of the 2002 Conference 15: 125.

[15] Diehl RL (2008) Acoustic and auditory phonetics: the adaptive design of speech sound systems. Philos Trans R Soc Lond, B, Biol Sci 363: 965–978. 17827108 10.1098/rstb.2007.2153 PMC2606790

[16] Eriksson JL, Villa AEP (2006) Learning of auditory equivalence classes for vowels by rats. Behav Processes 73: 348–359. 16997507 10.1016/j.beproc.2006.08.005

[17] Itskov PM, Vinnik E, Honey C, Schnupp J, Diamond ME (2012) Sound sensitivity of neurons in rat hippocampus during performance of a sound-guided task. J Neurophysiol 107: 1822–1834. 10.1152/jn.00404.2011 22219030 PMC3331670

[18] Engineer CT, Perez CA, Chen YH, Carraway RS, Reed AC, Shetake JA, et al (2008) Cortical activity patterns predict speech discrimination ability. Nat Neurosci 11: 603–608. 10.1038/nn.2109 18425123 PMC2951886

[19] Shadlen MN, Newsome WT (1995) Is there a signal in the noise? Curr Opin Neurobiol 5: 248–250. 7620314 10.1016/0959-4388(95)80033-6

[20] Parker AJ, Newsome WT (1998) Sense and the single neuron: probing the physiology of perception. Annu Rev Neurosci 21: 227–277. 9530497 10.1146/annurev.neuro.21.1.227

[21] Hastie T, Tibshirani R, Friedman J (2009) The Elements of Statistical Learning New York, NY: Springer Science & Business Media 1 pp.

[22] Paxinos G, Watson C (2007) The Rat Brain in Stereotaxic Coordinates. 1 pp. 10.1016/0165-0270(80)90021-7 6110810

[23] Chichilnisky EJ (2001) A simple white noise analysis of neuronal light responses. Network (Bristol, England) 12: 199–213. 11405422

[24] Rabinowitz NC, Willmore BDB, Schnupp JWH, King AJ (2011) Contrast gain control in auditory cortex. Neuron 70: 1178–1191. 10.1016/j.neuron.2011.04.030 21689603 PMC3133688

[25] Guo W, Chambers AR, Darrow KN, Hancock KE, Shinn-Cunningham BG, Polley DB (2012) Robustness of cortical topography across fields, laminae, anesthetic states, and neurophysiological signal types. Journal of Neuroscience 32: 9159–9172. 10.1523/JNEUROSCI.0065-12.2012 22764225 PMC3402176

[26] Bizley JK, Nodal FR, Nelken I, King AJ (2005) Functional organization of ferret auditory cortex. Cereb Cortex 15: 1637–1653. 15703254 10.1093/cercor/bhi042

[27] Sutter ML, Schreiner CE (1991) Physiology and topography of neurons with multipeaked tuning curves in cat primary auditory cortex. J Neurophysiol 65: 1207–1226. 1869913 10.1152/jn.1991.65.5.1207

[28] Hubel D, Wiesel T (1968) Receptive fields and functional architecture of monkey striate cortex. J Physiol (Lond). 10.1113/jphysiol.1968.sp008455 PMC1557912 4966457

[29] Barbary el A (1991) Auditory nerve of the normal and jaundiced rat. II. Frequency selectivity and two-tone rate suppression. Hear Res 54: 91–104. 1917720 10.1016/0378-5955(91)90139-z

[30] Ruggero MA, Temchin AN (2005) Unexceptional sharpness of frequency tuning in the human cochlea. Proc Natl Acad Sci USA 102: 18614–18619. 16344475 10.1073/pnas.0509323102 PMC1311742

[31] Syka J, Rybalko N, Brožek G, Jilek M (1996) Auditory frequency and intensity discrimination in pigmented rats. Hear Res 100: 107–113. 8922984 10.1016/0378-5955(96)00101-3

[32] Talwar SK, Gerstein GL (1998) Auditory frequency discrimination in the white rat. Hear Res 126: 135–150. 9872142 10.1016/s0378-5955(98)00162-2

[33] Bizley JK, Walker KMM, King AJ, Schnupp JWH (2010) Neural ensemble codes for stimulus periodicity in auditory cortex. Journal of Neuroscience 30: 5078–5091. 10.1523/JNEUROSCI.5475-09.2010 20371828 PMC2864913

[34] Chechik G, Anderson MJ, Bar Yosef O, Young ED, Tishby N, Nelken I (2006) Reduction of information redundancy in the ascending auditory pathway. Neuron 51: 359–368. 16880130 10.1016/j.neuron.2006.06.030

[35] Schnupp J (2006) Auditory filters, features, and redundant representations. Neuron 51: 278–280. 16880121 10.1016/j.neuron.2006.07.016

[36] Cohen MR, Kohn A (2011) Measuring and interpreting neuronal correlations. Nat Neurosci 14: 811–819. 10.1038/nn.2842 21709677 PMC3586814

[37] Harrington IA, Stecker GC, Macpherson EA, Middlebrooks JC (2008) Spatial sensitivity of neurons in the anterior, posterior, and primary fields of cat auditory cortex. Hear Res 240: 22–41. 10.1016/j.heares.2008.02.004 18359176 PMC2515616

[38] Szymanski FD, Rabinowitz NC, Magri C, Panzeri S, Schnupp JWH (2011) The laminar and temporal structure of stimulus information in the phase of field potentials of auditory cortex. Journal of Neuroscience 31: 15787–15801. 10.1523/JNEUROSCI.1416-11.2011 22049422 PMC6623019

[39] Harper NS, Mcalpine D (2004) Optimal neural population coding of an auditory spatial cue. Nature 430: 682–686. 15295602 10.1038/nature02768

[40] Aubie B, Sayegh R, Fremouw T, Covey E, Faure PA (2014) Decoding stimulus duration from neural responses in the auditory midbrain. J Neurophysiol. 10.1152/jn.00360.2014 PMC4233269 25122706

[41] Patterson RD, van Dinther R, Irino T (2007) The robustness of bio-acoustic communication and the role of normalization. Proc Int Congress on Acoustics: 1–6.

[42] Abeles M, Goldstein MH (1970) Functional architecture in cat primary auditory cortex: columnar organization and organization according to depth. J Neurophysiol 33: 172–187. 5411512 10.1152/jn.1970.33.1.172

[43] Merzenich MM, Knight PL, Roth GL (1975) Representation of cochlea within primary auditory cortex in the cat. J Neurophysiol 38: 231–249. 1092814 10.1152/jn.1975.38.2.231

[44] Clarey JC, Barone P, Imig TJ (1994) Functional organization of sound direction and sound pressure level in primary auditory cortex of the cat. J Neurophysiol 72: 2383–2405. 7884466 10.1152/jn.1994.72.5.2383

[45] Mendelson JR, Schreiner CE, Sutter ML, Grasse KL (1993) Functional topography of cat primary auditory cortex: responses to frequency-modulated sweeps. Experimental brain research Experimentelle Hirnforschung Experimentation cerebrale 94: 65–87. 8335076 10.1007/BF00230471

[46] Brugge JF, Merzenich MM (1973) Responses of neurons in auditory cortex of the macaque monkey to monaural and binaural stimulation. J Neurophysiol 36: 1138–1158. 4761724 10.1152/jn.1973.36.6.1138

[47] Middlebrooks J, Clock A, Xu L, Green D (1994) A panoramic code for sound location by cortical neurons. Science 264: 842–844. 8171339 10.1126/science.8171339

[48] Gilbert CD (1977) Laminar differences in receptive field properties of cells in cat primary visual cortex. J Physiol (Lond) 268: 391–421. 874916 10.1113/jphysiol.1977.sp011863 PMC1283670

[49] Linden JF, Schreiner CE (2003) Columnar transformations in auditory cortex? A comparison to visual and somatosensory cortices. Cereb Cortex 13: 83–89. 12466219 10.1093/cercor/13.1.83

[50] Phillips DP, Irvine DR (1981) Responses of single neurons in physiologically defined primary auditory cortex (AI) of the cat: frequency tuning and responses to intensity. J Neurophysiol 45: 48–58. 7205344 10.1152/jn.1981.45.1.48

[51] Sugimoto S, Sakurada M, Horikawa J, Taniguchi I (1997) The columnar and layer-specific response properties of neurons in the primary auditory cortex of Mongolian gerbils. Hear Res 112: 175–185. 9367240 10.1016/s0378-5955(97)00119-6

[52] Szymanski FD, Garcia-Lazaro JA, Schnupp JWH (2009) Current source density profiles of stimulus-specific adaptation in rat auditory cortex. J Neurophysiol 102: 1483–1490. 10.1152/jn.00240.2009 19571199

[53] Winkowski DE, Kanold PO (2013) Laminar transformation of frequency organization in auditory cortex. Journal of Neuroscience 33: 1498–1508. 10.1523/JNEUROSCI.3101-12.2013 23345224 PMC3783029

